# Random networks of core-shell-like Cu-Cu_2_O/CuO nanowires as surface plasmon resonance-enhanced sensors

**DOI:** 10.1038/s41598-018-23119-6

**Published:** 2018-03-16

**Authors:** Rashad Hajimammadov, Alexander Bykov, Alexey Popov, Koppany L. Juhasz, Gabriela S. Lorite, Melinda Mohl, Akos Kukovecz, Mika Huuhtanen, Krisztian Kordas

**Affiliations:** 1 Microelectronics Research Unit, Faculty of Information Technology and Electrical Engineering, University of Oulu, P.O. Box 4500, 90570 Oulu, Finland; 2 Optoelectronics and Measurement Techniques Research Unit, Faculty of Information Technology and Electrical Engineering, University of Oulu, P.O. Box 4500, 90570 Oulu, Finland; 3 Department of Applied and Environmental Chemistry, University of Szeged, Rerrich Bela ter 1, Szeged, 6720 Hungary; 4 Environmental and Chemical Engineering Research Unit, Faculty of Technology, University of Oulu, P.O. Box 4300, 90570 Oulu, Finland

## Abstract

The rapid oxide formation on pristine unprotected copper surfaces limits the direct application of Cu nanomaterials in electronics and sensor assemblies with physical contacts. However, it is not clear whether the growing cuprous (Cu_2_O) and cupric oxides (CuO) and the formation of core-shell-like Cu-Cu_2_O/CuO nanowires would cause any compromise for non-contact optical measurements, where light absorption and subsequent charge oscillation and separation take place such as those in surface plasmon-assisted and photocatalytic processes, respectively. Therefore, we analyze how the surface potential of hydrothermally synthetized copper nanowires changes as a function of time in ambient conditions using Kelvin probe force microscopy in dark and under light illumination to reveal charge accumulation on the nanowires and on the supporting gold substrate. Further, we perform finite element modeling of the optical absorption to predict plasmonic behavior of the nanostructures. The results suggest that the core-shell-like Cu-Cu_2_O/CuO nanowires may be useful both in photocatalytic and in surface plasmon-enhanced processes. Here, by exploiting the latter, we show that regardless of the native surface oxide formation, random networks of the nanowires on gold substrates work as excellent amplification media for surface-enhanced Raman spectroscopy as demonstrated in sensing of Rhodamine 6G dye molecules.

## Introduction

Since the Bronze Age, copper has been widely used in everyday life in different areas of industry for numerous purposes. Its highest thermal and electrical conductivity among commonly available materials, as well as a relatively low price and high abundance in the lithosphere, made copper one of the most important technical materials. However, it has a main and unavoidable drawback, rapid oxidation, when exposed to ambient oxygen. The surface oxidation of copper results in reduced electrical and thermal conductivity and increased contact resistance. In the attempt to limit and avoid the negative effects of oxidation, different methods have been proposed, such as wrapping copper with either polymers or other coatings^1,2^ and selective removal of surface oxides by acid etching or plasma cleaning has been applied^3^. On the other hand, one may take advantage of oxidation in photovoltaics^4^, catalysis^5^ and gas sensing^6^. For these applications, copper oxides are usually synthesized on metallic copper films by thermal annealing, which is commonly accepted as a fast, cheap and efficient method for the preparation of homogeneous oxide layers. As copper is oxidized at a slower rate at room temperature than at elevated temperatures, the natively formed oxides can be an interesting case to study where surface composition of the metal changes gradually, altering the electrical, optical and chemical properties of the material.

When oxidized, copper forms two p-type oxides, cuprous (Cu_2_O) and cupric oxide (CuO) with corresponding band gaps of 2.2 eV and 1.4 eV, respectively. Because of their narrow band gap, both oxides are sensitive to visible light allowing inter-band excitation of electrons from the valence to conduction band by illumination. Consequently, one may expect that under various illumination conditions and/or in different chemical environments, the surface charge density changes and can be detected, e.g. by Kelvin probe force microscopy (KPFM), as it was demonstrated for other metal-semiconductor systems^7,8^. Furthermore, similar to gold and silver metal nanostructures^9^, also copper shows reasonable plasmonic absorption in the visible spectrum^10,11^, thus it is expected to be suitable as an amplification medium in surface-enhanced Raman spectroscopy (SERS) analysis of various compounds.

In this work, we used this opportunity to reveal the plasmonic properties of copper nanowires with growing native oxides on the surface. The results indicate that despite the formed oxides Cu nanowires are suitable for visible light induced chemical sensing using SERS method thus expanding the use of these easy to synthesize and affordable materials in the future.

**Figure 1:**
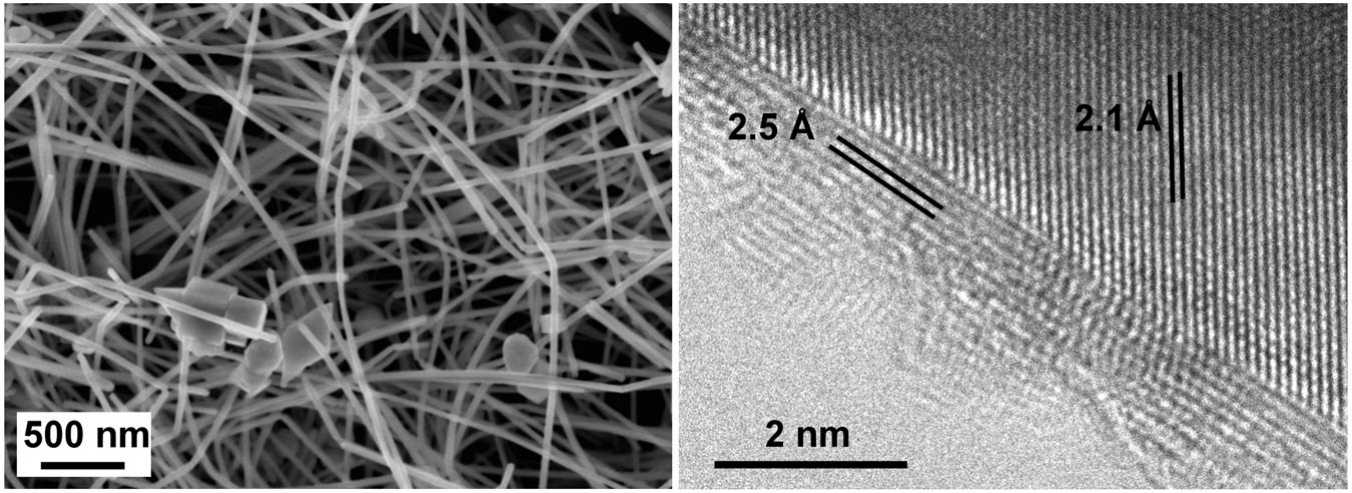
SEM and HRTEM images of copper nanowires. In the right panel the interplanar spacing values of 2.1 Å and 2.5 Å correspond to the (111) planes of Cu and Cu_2_O, respectively.

Scanning electron microscopy (SEM) analysis shows that the synthesized copper nanowires have a large aspect ratio; the diameter of the nanowires is between 20–100 nm (with an average of ~50 nm) and their length can reach up to 50 µm (Fig. 1). The high-resolution transmission electron microscopy (HRTEM) image of a selected nanowire exhibits the (110) growth direction and the formation of an oxide layer of varying thickness on the nanowire surface caused by a short exposure (a few minutes that are needed for sample preparation) to atmospheric oxygen. Interatomic spacing values measured on the HRTEM image (Fig. 1) were found to be 2.1 Å and 2.5 Å, and were attributed to the (111) planes of Cu and Cu_2_O, respectively. The formation of Cu_2_O on the surface of the nanowire in early periods of oxygen exposure matches well our previous report^12^, where the oxidation mechanism of nanowires was studied for a period of 0–30 days.

**Figure 2:**
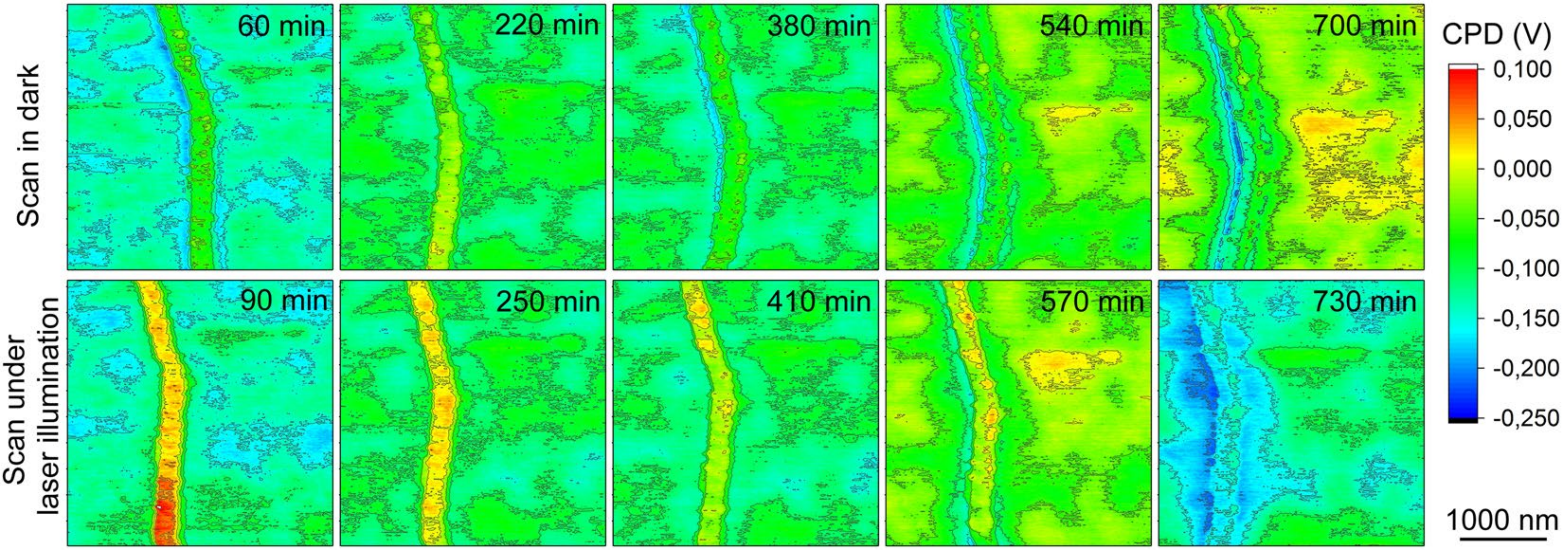
Color map surface potential measured for an oxidizing copper nanowire in contact with a gold surface. The surface potential maps recorded in dark (upper row) and under laser illumination (lower row) on the same nanowire. The time values in the corners of the panels indicate time interval of oxidation the nanowire has gone through. Please note, the sequence of measurement alternates the dark and laser-illuminated measurements.

For analyzing any work function changes caused by the ambient conditions in time, Kelvin probe force microscopy (KPFM) mapping of Cu nanowires deposited on an Au substrate was performed^13^. The change of work function for the nanowire as a function of oxidation period can be observed in Fig. 2 (upper panels). For short term (1 hour) exposure to atmospheric air, our results reveal a contact potential difference (CPD) of 100 mV between the nanowire and the Au surface as shown in Fig. 2 (first scan in dark, oxidation period of 1 hour). This result is consistent with the negligible work function difference between copper oxides (5.3 eV for Cu_2_O and 5.2 eV for CuO) and sputtered Au (5.3 eV)^14,15^. Our time-dependent measurements clearly show a continuous change of the surface potential measured on the nanowire. The change may be caused by the progress of oxidation and gradual development of a continuous oxide shell on the metallic core^16^. The surface potential change is also observed when the Cu nanowire is illuminated with a laser beam as shown in Fig. 2 (lower panels). Nevertheless, it is also evident that the CPD shifts to higher values when illuminated, and recovers to a value close to the initial one when scanned again in the dark (Fig. 2) indicating a reversible light response of the partially oxidized Cu nanowire located on the Au substrate.

The observed shifts of the surface potential near the nanowire can be explained by at least two mechanisms responsible for charge generation and separation. The first mechanism is based on a photo-generation of charge carriers in semiconducting metal oxides, with subsequent electron or hole injection to the metals in contact with n-type or p-type oxides, respectively. The scheme is analogous to photocatalytic composites of semiconducting support and metal nanoparticles, which form nano-sized Schottky junctions^17,18^. After injection of holes from the valence band of the semiconducting Cu_2_O shell to the metals in contact (i.e. Cu core, Pt tip and Au substrate) the surplus charge in the Cu core faces a significant barrier (~0.8 eV) to return to Cu_2_O. Hence, an efficient separation of the photo-generated electron-hole pairs takes place (Figure S1) thus modulating the surface potential upon light exposure. Note, while the holes face a barrier to inject from Cu_2_O back to Cu, the Pt-Cu_2_O contact is ohmic, whereas the Au-Cu_2_O has a very low barrier of ~0.2 eV, thus not expected to participate in rectification. It is important to point out that in our experiments, the photon energy of the green laser (λ = 552 nm) we applied in the KPFM measurements is ~2.2 eV, which is practically the same as the band gap of Cu_2_O. Therefore, interband electron transition may take place only between the top edge of the valence band and the bottom edge of the conduction band. Since the density of states near the band edges is typically low, we expect only weak optical absorption and limited photo-generation. Accordingly, in principle, we may observe surface potential shift caused by the photo-generated and then separated carriers; this process is expected to have limited effects.

Another mechanism that can result in charging of the surface is associated with hot electrons generated in the metal via plasmonic processes. After fast optical absorption in metals, several events can take place depending on the energy of incoming photon and the type and geometry of the metal. High-energy ultraviolet photons, whose energy exceed the work function of metals can induce photoelectrons. When the photon energy is lower, as by visible or infrared irradiation, the localized surface plasmons decay quickly through intraband or interband excitation of electrons that thermalize (via electron-electron and electron-phonon interactions) unless we trap and utilize those. One approach for trapping the charge is possible by bringing the metals in contact with semiconductors to form rectifying i.e. Schottky contacts. After optical absorption, electrons that are excited over the Fermi level can inject to the conduction band of an n-type semiconductor provided their energy is higher than the Schottky barrier (while leaving behind holes in the metal), thus inducing charge separation. The picture in our case is somewhat different, since Cu_2_O is a p-type semiconductor, therefore electrons can inject back to the metal from the conduction band of the oxide. In fact, rectification of holes takes place as those can inject to the valence band of Cu_2_O. The energy of the holes in Cu has been calculated to be more than an 1 eV below the Fermi level, when illuminating the metal surface with visible photons (400 nm and 700 nm)^19^. After injection to the semiconductor through the Schottky barrier (~0.8 eV), these holes face a barrier of ~0.6 eV upon returning to Cu^20–23^.

It is worth mentioning here that experiments using an IR laser (λ = 980 nm) to illuminate the nanowires during KPFM analysis showed no change of the surface potential as compared to the dark condition (Figure S2) due to the low photon energy (~1.2 eV). This is in a reasonable agreement with our proposed mechanisms related to the need of electron transition from the valence band and/or from the Fermi level of Cu to the conduction band in Cu_2_O (photo-generation and/or plasmonic process, respectively).

**Figure 3:**
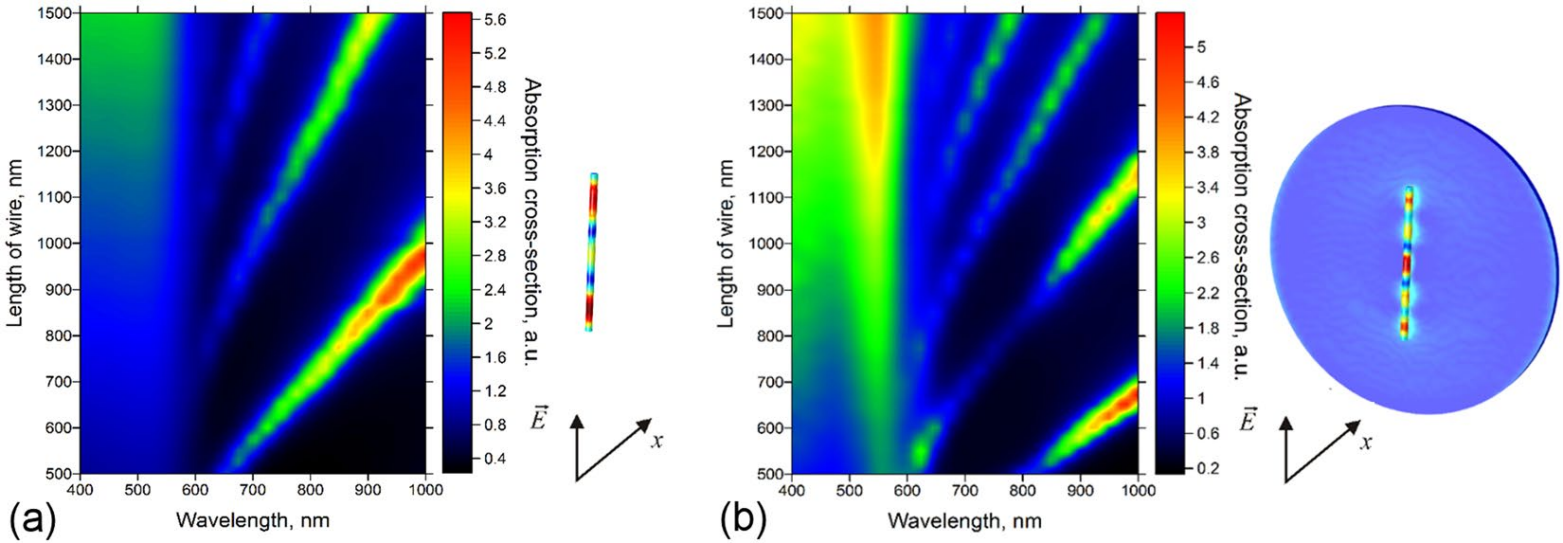
2D color maps of calculated absorption cross-section at different nanowire lengths in the spectral range of 400–1000 nm for (**a**) an isolated Cu nanowire in air and (**b**) on the surface of Au.

To explore further plasmonic absorption that may potentially be responsible for light-induced processes in the nanowires, we simulate the optical absorption (and also the local field enhancement as shown in the video files in Video S1, S2 and S3) for a nanowire with a diameter of 50 nm, where the incident electromagnetic wave is propagating perpendicular to the wire and thus its electric field vector is parallel to the wire. (Note: When the wave propagation is parallel to the nanowire, i.e. the electric field vector is perpendicular, we do not observe plasmonic peaks as displayed in Figure S3 being in a good agreement to previous reports for Ag and Au nanowires)^24,25^. We find that the absorption of the radiation with the wavelength shorter than 600 nm grows with the increasing length of the nanowire as displayed in Fig. 3(a), which is explained by the linear growth of the nanowire volume with the increase of the wire length. Peaks of plasmon resonance appear at longer wavelengths, whose position depends on the length of the nanowire, which is typical for longitudinal plasmon modes^26–28^. Simulations for the Cu nanowire on a Au substrate show an apparent difference in the absorption cross-section as displayed in Fig. 3(b). Below 600 nm wavelength, a pronounced increase of the absorption cross-section is observed because of the plasmon-resonant gold substrate, which enhances the electric field and also leads to its increased absorption by the Cu nanowire. At longer wavelengths, additional resonant modes appear. Interestingly, in the case of longer nanowires (simulated for a length of 5 µm), the ones on the Au substrate show a strong absorption peak around 520 nm wavelength, typical for plasmon resonance of Au (Figure S4).

**Figure 4:**
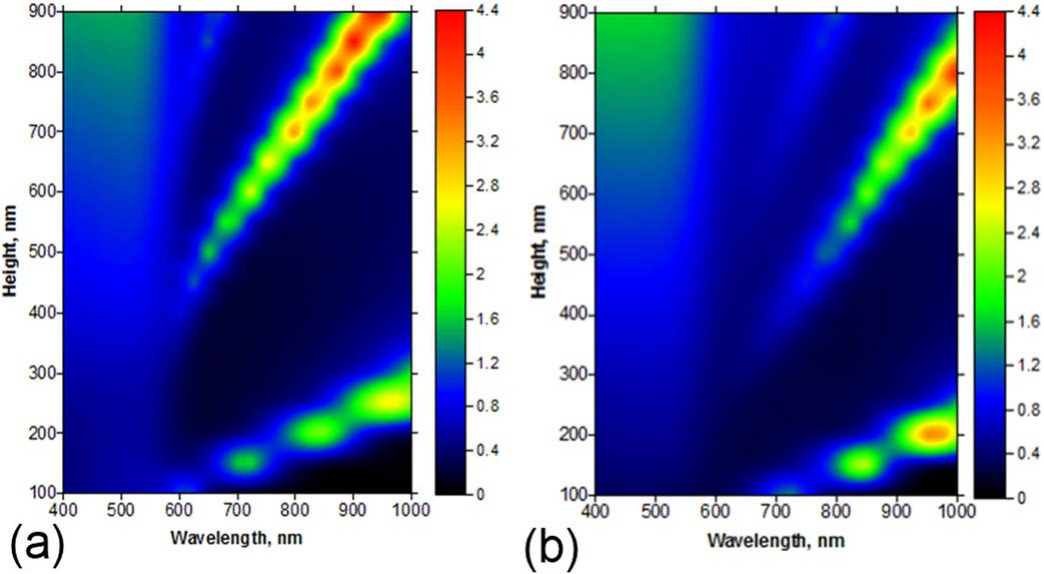
Simulated absorption cross-section of (**a**) a pure Cu nanowire and (**b**) with a 5 nm CuO shell as functions of the wavelength and nanowire length. The nanowire thickness is 50 nm.

To reveal the influence of the copper oxide layer grown on the surface of the nanowire, we also performed simulations for isolated nanowires with different oxide thickness (Fig. 4 and Figure S5). The appearance of surface oxide results in a red shift proportional to the increase of oxide thickness. The red shift may be explained by the increased aspect ratio of the metal nanowire as well as by the changed dielectric behavior of the medium (i.e., copper oxides instead of air) surrounding the metal.

**Figure 5:**
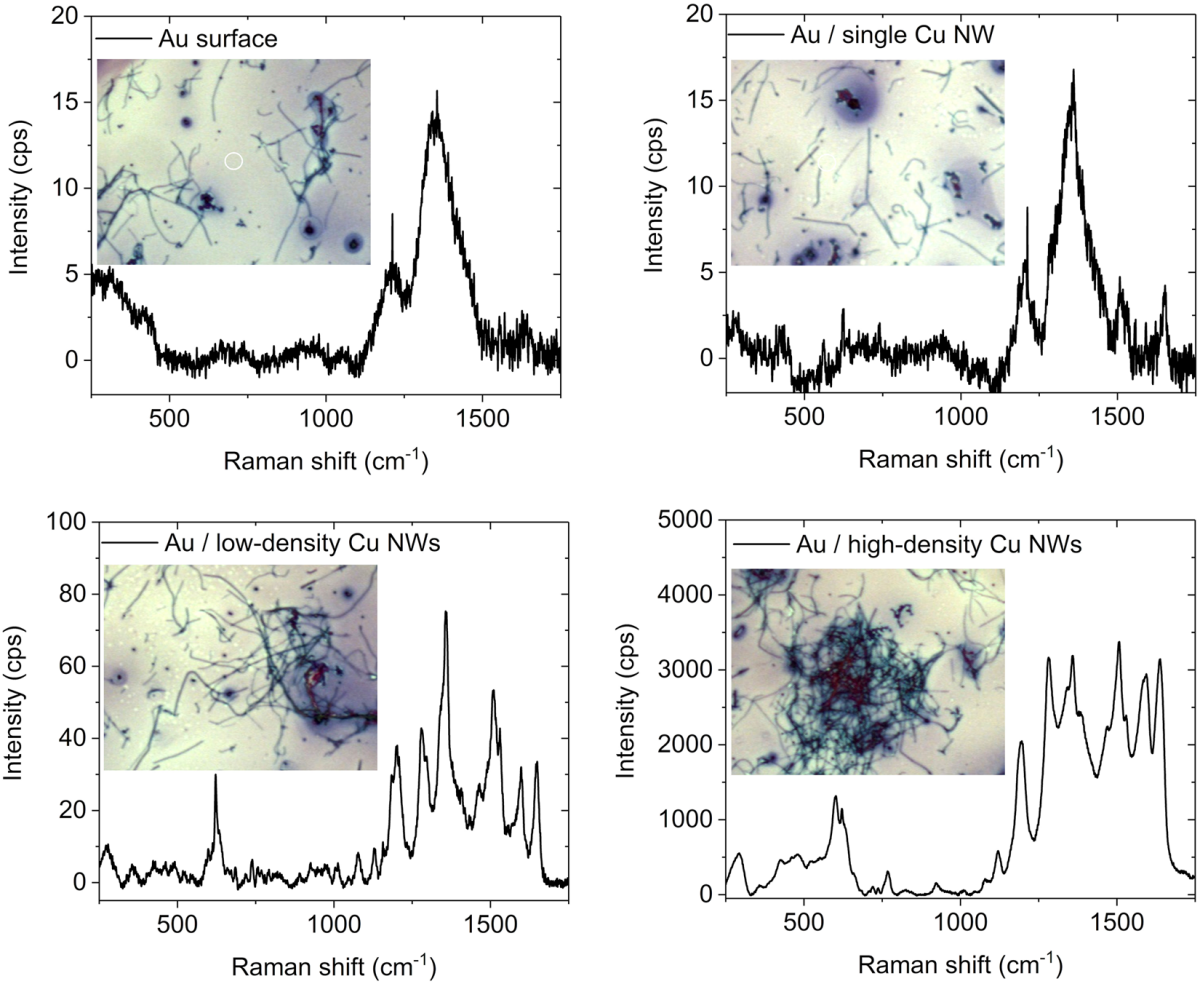
SERS measurements with random networks of the nanowires. Optical images and corresponding Raman spectra (in the locations of the center of the micrograph as highlighted with white circles) of R6G on Au surface with different Cu nanowire densities. Experimental parameters: 10 mW laser power, 50× objective, slit aperture 50 µm, exposition time 3 s, sampling 32×.

Further, to take advantage of the plasmonic behavior of the nanowires, we explored this function further to be used as a sensing amplification medium for surface-plasmon enhanced Raman spectroscopy^29^. For this, a well-studied organic dye, Rhodamine 6 G (R6G) was selected as a model compound. Cu nanowires suspended in ethanol were drop cast on a chip having Au/Cr coating of 200 nm thickness. After drying, we deposited a drop of R6G solution on the surface and let the sample dry again in ambient conditions before starting the Raman measurements (excitation at 780 nm, nominal beam waist of 1 µm in the laser focal spot). As shown in Fig. 5, the SERS intensities are highly dependent on the number density of nanowires on the Au surface, as it is expected because of the increased absorption as well as Raman scattering cross-sections of the amplification medium. Without nanowires, the overall peak intensities are very low, and it is difficult to resolve the characteristic Raman vibration modes of the dye (in-plane and out-of-plane xanthene ring deformation at 609 cm^−1^, out-of-plane C–H bending at 775 cm^−1^, in-plane xanthene ring deformation (C–H bending, N–H bending) at 1187 cm^−1^, in-plane xanthene ring breathing (N–H bending, CH_2_ wagging) at 1305 cm^−1^, in-plane xanthene ring stretching (C–H bending) at 1360 cm^−1^, in-plane xanthene ring stretching (C–H bending, N–H bending and C–N stretching) 1506 cm^−1^, in-plane xanthene ring stretching (N–H bending) 1572 cm^−1^, and in-plane xanthene ring stretching (C–H bending) at 1648 cm^−1^)^2,30,31^. When acquiring the spectra in locations having at least a few nanowires, we observed an increased SERS signal, and better-resolved peaks in the spectra. In the locations with the largest nanowire number density, the spectra are well-resolved and show nearly three orders of magnitude amplification compared to the pure Au substrate, which is remarkable considering that no specific SERS substrate with any lithographic pattern definition was used.

At least three different interpretations of the enhancement factor (EF) are present in the SERS literature: (a) the single molecule enhancement factor, (b) the SERS substrate enhancement factor and (c) the analytical enhancement factor^32^. The single molecule EF is unaffected by the morphology of the substrate because it is determined by the Raman tensor of the analyte and the local field. However, the enhanced Raman intensity measured in our experiments is the sum of all scattering events occurring in the laser spot volume. Therefore, it is the structure of the nanowire network in this volume that determines the intensities reported in Fig. 5. Surface enhancement occurs only when the analyte is adsorbed on hotspots. It is generally agreed that wire–wire crossings generate such hotspots, therefore, higher overall enhancement is usually expected for denser nanowire agglomerates. The actual number of wire–wire contacts is a nonlinearly increasing function of the solid contents of the probed volume^33,34^, which qualitatively explains the observed Raman intensity differences in Fig. 5. It is worth noting that the existence of this correlation between Cu nanowire density and Raman intensity is not trivial at all. Sparse nanowire networks have been shown to exhibit counter-intuitive electrical conductivity characteristics^35^, and Clayton *et al*. have recently reported that the SERS spectra of 4-mercaptopyridine was not significantly influenced when the measurement was moved from a single silver nanowire to two crossed wires^36^.

**Figure 6:**
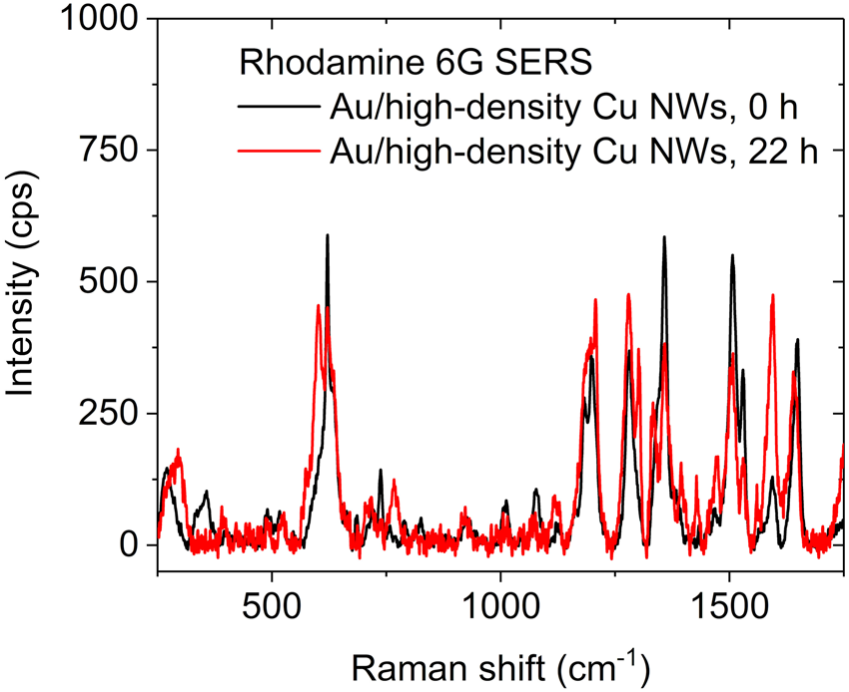
Effect of oxidation of Cu nanowires on SERS signal intensity. Raman spectra of Rh6G on a Au substrate with high number density of Cu nanowires measured using fresh and oxidized substrates. Experimental parameters: 10 mW laser power, 50× objective, slit aperture 25 µm, exposure time 2 s, sampling 20×.

One may expect a significant change of the SERS amplification as the test specimen is exposed to air due to the oxidation of Cu nanowires. However, we found no considerable differences between the R6G Raman spectra collected immediately after sample preparation and the next day (Fig. 6), which makes the Cu nanowires quite promising materials for such applications. The main difference between the spectra is in the I_1572_/I_1651_ intensity ratio. A possible explanation can be offered based on the different origin of the peaks. Even though all three peaks in this region (1506 cm^−1^, 1572 cm^−1^ and 1651 cm^−1^) are related to xanthane ring stretching vibrations^31^, the 1572 cm^−1^ feature is also linked to in-plane N–H bending, whereas the other two have less N–H bending and more C–H bending contributions. Considering the well-known affinity of Cu(I) and Cu(II) species to nitrogen-containing complexing agents, it seems reasonable to assume that the 1572 cm^−1^ peak would be more affected by the oxidation of the metallic copper substrate than those with less pronounced contributions from nitrogen-related bonds. The partial oxidation of the substrate could create adsorption sites where the Rh6G molecule is preferentially attached using its secondary amine function, which in turn would increase the polarizability of the N–H bond and thus increase the Raman cross-section of the mode contributing to the 1572 cm^−1^ peak. The general feasibility of this hypothesis can be confirmed by noting that Lin and co-workers have observed a similar disproportional scaling (with Rh6G concentration) in the same spectral region when studying the SERS of Rh6G using a single Cu_2_O superstructure particle as a substrate^37^, and Wang *et al*. have observed a change of the adsorption mode Rh6G when fine-tuning the surface properties of a TiO_2_ substrate^38^.

In conclusion, the oxidation of Cu nanowires in ambient conditions is visible by Kelvin-probe force microscopy measurements. The surface potential of the oxidizing nanowires is greatly depending not only on the oxidation time but also on the illumination conditions. The detected reversible light-induced charge-discharge behavior suggests photocatalytic applications of the core-shell-like Cu-Cu_2_O/CuO nanowires. On the other hand, the nanowires display surface-plasmon absorption as revealed by finite-element modeling. Interestingly, the growth of native oxide on the surface seems to have a negligible effect on the plasmonic behavior of Cu nanowires making these materials suitable for surface-enhanced Raman spectroscopy analyses of adsorbed compounds (Rhodamine 6G in our studies). The results reported here may expand the list of use of Cu nanowires and pave the way for chemical catalyst as well as for sensing applications.

## Materials and Methods

A simple hydrothermal method^39^ was used to synthesize copper nanowires. Copper chloride dihydrate CuCl_2_·2H_2_O, D-glucose and hexadecylamine (Sigma Aldrich) were used as received without further purification. Copper chloride dihydrate was dissolved in deionized water then D-glucose and hexadecylamine were added slowly under vigorous stirring. After 5 hours of stirring a light blue turbid dispersion formed and was placed into a Teflon® lined autoclave (Parr Instrument, Model 4525, 1000 mL) and heated to 393 K. After 24 hours of reaction and spontaneous cooling, the obtained brown dispersion was collected and centrifuged at 2500 rpm (990 RCF) for 15 min. The supernatant was discarded and the reddish solid was redispersed in DI water, n-hexane and ethanol to clean the product. The centrifuging, decantation and redispersion cycle was repeated three times in each solvent, and finally, the obtained copper nanowires were kept under n-hexane or ethanol until further analyses to prevent them from oxidation.

Field emission scanning electron microscopy (FESEM, Zeiss Sigma) was used to determine the shape and the size of the synthesized nanowires. The starting oxide formation on the nanowires was assessed by high-resolution TEM (JEOL 2200 FS). Dispersion of copper nanowires in ethanol was drop cast on Ni grids with holey carbon. Mapping of surface potential difference was performed by using atomic force microscopy (AFM, MultiMode 8, Nanoscope V, Bruker, USA) in Peak Force-KPFM mode^40^. Small drops of low concentration copper nanowire dispersion in hexane were cast on 50 nm (111) gold sputtered silicon wafer chip preliminarily grounded with a silver paint on a magnetic steel disk. Pt coated Si probes (NSC18/Pt, MicroMash, Tallin, Estonia) with calibrated 2.91 and 2.59 N/m spring constant and tip radius of 30 nm were used for scanning a sample area of 3.12 µm × 3.12 µm (256 × 256 lines) with a scan rate of 0.220 Hz. Mapping of surface potential was carried out in two passes, where the first scan defined the surface morphology and the second scan, driven 100 nm high over the sample, detected surface potential change. A visible laser (Coherent, OBIS) with 552 nm wavelength operated at 20 mW power was used to illuminate the surface of a Au coated chip under a low angle of incidence to ensure the coverage of the whole area and eliminating the shadow that could possibly appear from the cantilever. Scanning sequences were performed by alternating dark and illuminated conditions.

Simulations of the electromagnetic wave interaction with a Cu wire and calculation of corresponding absorption cross-sections were performed using RF, Frequency Domain module of COMSOL 5.3 software. The developed model provides a convenient way of computing plasmon resonance parameters for arbitrary-shaped nano/micro-particles. As the input parameters, the model utilizes the values of the real and imaginary part of the refractive index of the particles. In this study, the properties of Cu^41^ and Au^42^ were used. As a result, the developed model provides the absorption cross-section of the considered particle as well as the spatial distribution of the scattered radiation and the electromagnetic field distribution on the surface of the particle. The developed model was validated by comparison of the obtained solution for a Cu sphere of 100 nm dimeter with the corresponding solution provided by the Mie theory. Excellent agreement was observed for the calculated parameters.

For SERS measurements, the samples were prepared as follows: Au/Cr coated Si wafers (SPI Supplies) were laser cut to a size of 8 × 8 mm^2^ chips, and a droplet of Cu nanowire suspension (in ethanol) was applied on the surface. After drying, aqueous Rhodamine 6 G solution (10^−3^ M using a powder of ~95% purity from Sigma Aldrich) of 10 µL volume was deposited on the surface and then dried at ambient conditions before starting the Raman measurements (Thermo Scientific DXR Raman instrument equipped with a high-resolution grid, wavelength: 780 nm, power: 10 mW, MPlan 50× objective, slit apertures with 50 and 25 µm opening).

### Data availability statement

All data generated or analyzed during this study are included in this published article (and its Supplementary Information files).

## Electronic supplementary material

supplementary information

Video S1

Video S2

Video S3
