# Supplementary material for: Random networks of core-shell-like Cu-Cu2O/CuO nanowires as surface plasmon resonance-enhanced sensors
Source: Sci Rep. 2018 Mar 16;8:4708. doi: 10.1038/s41598-018-23119-6 (PMC5856813; doi:10.1038/s41598-018-23119-6)
Supplement: Supplementary file 1 — supplementary information [file 41598_2018_23119_MOESM1_ESM.docx]

**Supporting information to *Random networks of core-shell-like Cu-Cu_2_O/CuO nanowires as surface plasmon resonance-enhanced sensors* by Hajimammadov et al.**

(a)
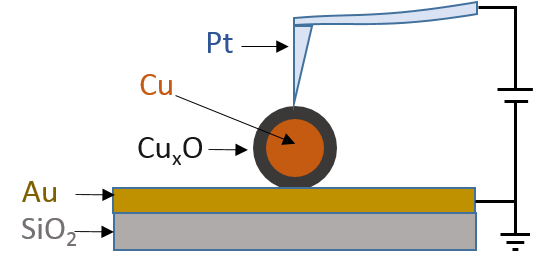


(b)
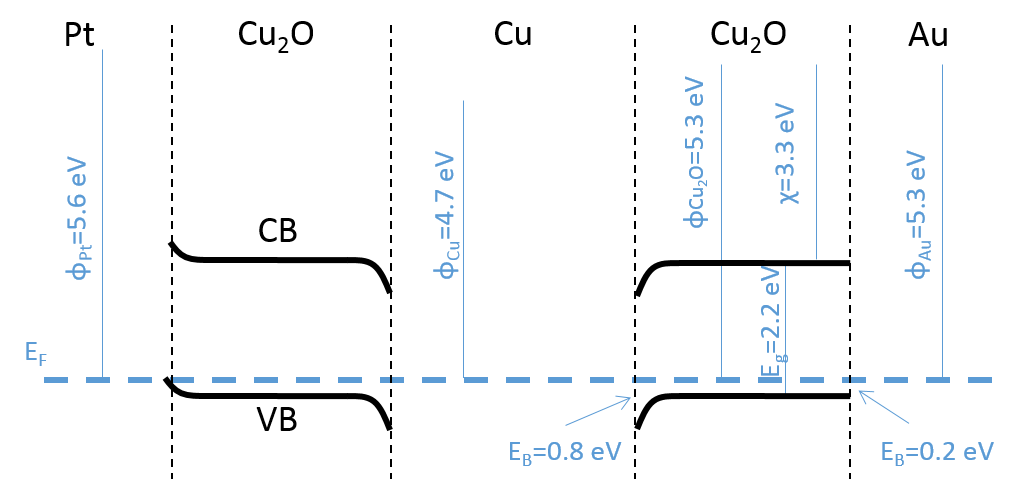


**Figure S1** (a) Schematics of oxide shell/metal core nanowire contact potential difference (CPD) mapping; (b) band diagram of oxide covered copper when in contact with Au substrate and Pt probe.


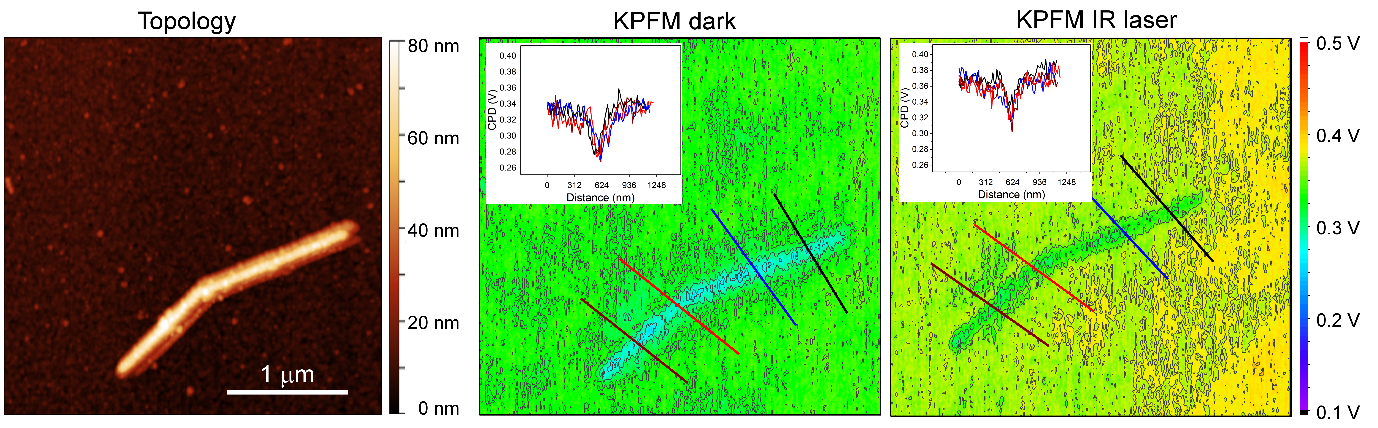


**Figure S2** Topology (left panel) and KPFM maps of a nanowire in dark (center panel) and under illumination using an IR laser having wavelength of 980 nm. The IR laser photons (~1.2 eV) have no sufficient energy to excite electrons from the valence band of Cu_2_O to the conduction band (E_g_~2.2 eV), thus we can rule out photogeneration, and consequently we are not expecting the change of surface potential. When considering hot electrons from the plasmonic process on the surface of the Cu core, the electrons shall have 1.4 eV energy to reach the edge of the conduction band in Cu_2_O, which is higher again than the energy of incoming photons. Accordingly, the IR photons will not be able to influence the surface potential in this process either.


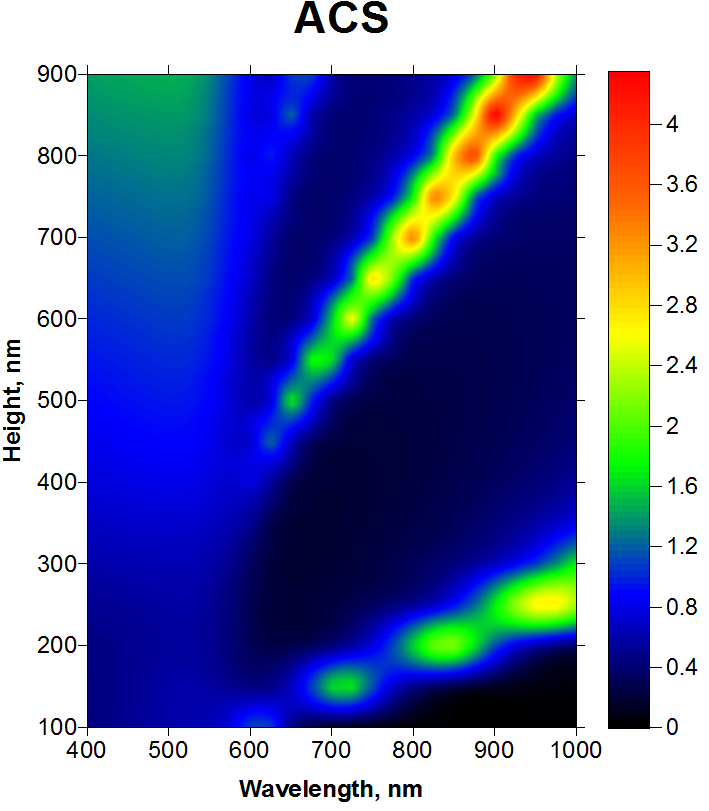

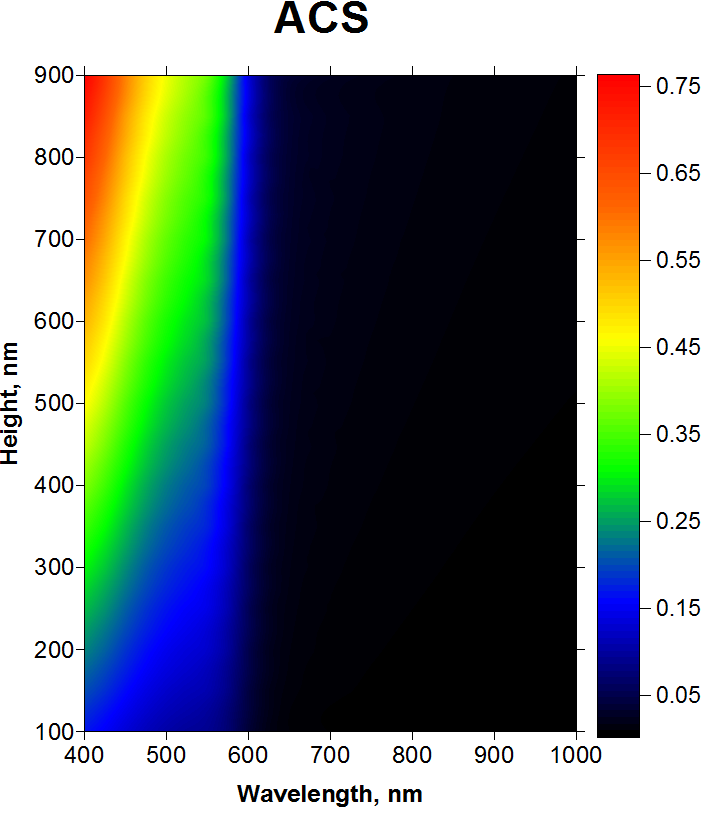


**Figure S3** Simulated absorption cross section of Cu wire in the case of parallel (left) and perpendicular (right) orientation of wire relative to the polarization of the incident electric field. plasmon peaks in the absorption cross section appear when the incident electric field polarization is parallel to the wire. Similar observation was reported earlier (M. Song, et al., Opt. Express, 20, 22290-22297 (2012) and R. Takahata, et al. J. Am. Chem. Soc., 136, 8489–8491 (2014).) for Ag and Au wires of comparable aspect ratio.




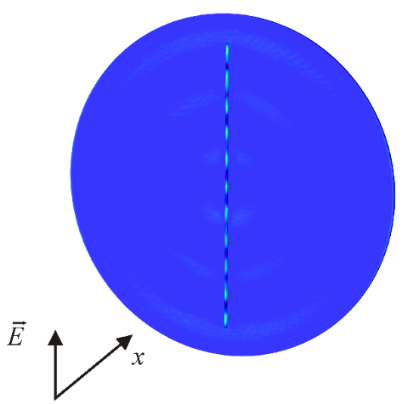


**Figure S4.** Spectral dependencies of the absorption cross-section for an isolated single Cu wire of 50 nm diameter and length of 5000 nm (red circles) and for the same wire in the presence of Au substrate with diameter of 6000 nm (blue squares).


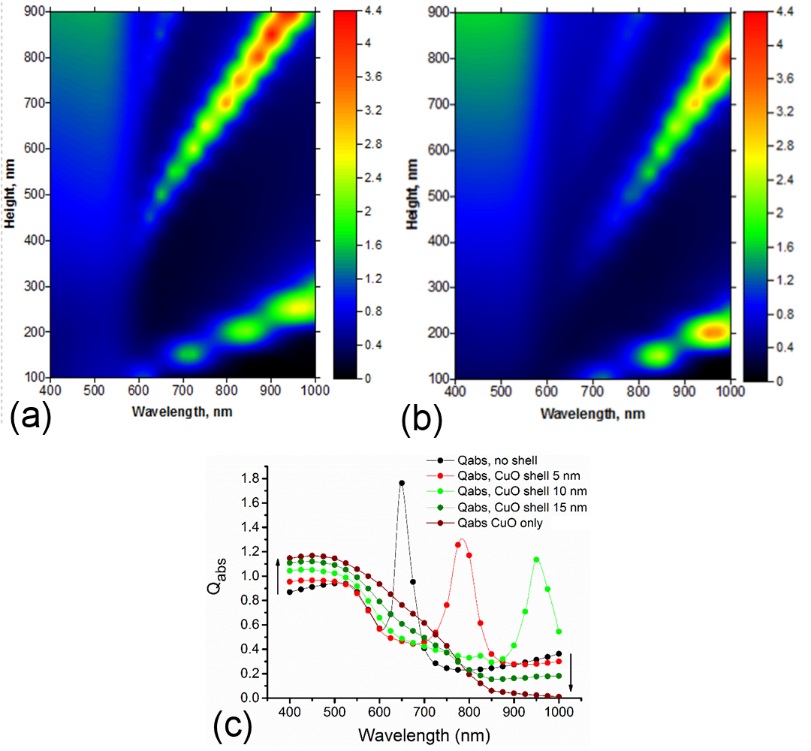


**Figure S5.** Simulated absorption cross section of a Cu NW of 500 nm length and diameter of 50 nm with and without CuO shell of different thickness.
